# Neuroprotective effects of alisol A 24-acetate on cerebral ischaemia–reperfusion injury are mediated by regulating the PI3K/AKT pathway

**DOI:** 10.1186/s12974-022-02392-3

**Published:** 2022-02-07

**Authors:** Taotao Lu, Huihong Li, Yangjie Zhou, Wei Wei, Linlin Ding, Zengtu Zhan, Weilin Liu, Jing Tao, Xiehua Xue

**Affiliations:** 1 The Affiliated Rehabilitation Hospital, Fujian University of Traditional Chinese Medicine, No. 13 Hudongzhi Road, Fuzhou City, 350003 Fujian Province China; 2 College of Rehabilitation Medicine, Fujian University of Traditional Chinese Medicine, Fuzhou, 350112 China; 3 Fujian Key Laboratory of Rehabilitation Techniques, Fuzhou, 350112 China

**Keywords:** GCI/R, Alisol A 24-acetate, PI3K/AKT, inflammation, Apoptosis

## Abstract

**Background:**

Neuroinflammation and apoptosis are involved in the pathogenesis of ischaemic stroke. Alisol A 24-acetate (24A) exerts a strong inhibitory effect on inflammation and cell apoptosis. The neuroprotective effect of 24A on global cerebral ischaemia/reperfusion (GCI/R) injury remains unclear.

**Methods:**

GCI/R mice were used to investigate the neuroprotective effect of 24A. Modified neurological deficit scores, Morris water maze and object recognition tests were used to evaluate behaviours. Metabolism in brain regions was detected using magnetic resonance spectroscopy (MRS), and changes in microglia, astrocytes and neurons were detected. Inflammation and apoptosis were measured.

**Results:**

The results showed that 24A suppressed neurological deficits scores and improved GCI/R induced cognitive dysfunction. It was also observed that 24A could alleviate neuroinflammation, which manifested as 24A inhibited microglia and astrocytes proliferation, downregulated the expression of interleukin (IL)-1β, tumor necrosis factor (TNF)-α, and inducible nitric oxide synthase (iNOS) in the GCI/R mice brain. The apoptosis of neurons reduced, and dendritic spines of hippocampal neurons increased in the presence of 24A. In addition, 24A could up-regulate the expression of phosphorylated phosphoinositide 3-kinases (p-PI3K) and phosphorylated protein kinase B (p-AKT) in GCI/R mice brain, and all the morphological, neurological, and biochemical changes of 24A treatment were abolished by the application of PI3K/AKT pathway inhibitor LY294002.

**Conclusions:**

Taken together, our study indicated that 24A alleviated GCI/R injury by inhibiting neuroinflammation and apoptosis through the regulation of the PI3K/AKT pathway.

**Supplementary Information:**

The online version contains supplementary material available at 10.1186/s12974-022-02392-3.

## Introduction

Ischaemic stroke results in damage to the brain function by altering the blood supply and induces a series of neurological symptoms, which seriously endanger people's health. Cerebral ischaemia–reperfusion (CI/R) injury caused by cerebrovascular recanalization is considered a serious problem in the treatment of ischaemic stroke [1, 2]. CI/R is a complex pathophysiological process involving various mechanisms, such as the release of excitatory amino acids, oxidative stress, apoptosis and inflammation. Accumulating evidence indicates that neuroinflammation and apoptosis are involved in the pathogenesis of ischaemic stroke. However, numerous neuroprotective drugs have failed to show benefits in the treatment of ischaemia–reperfusion (I/R) after ischaemic stroke, and thus the identification of new treatments is urgently needed.

Alisol A 24-acetate (24A), a protostane-type tetracyclic triterpenoid, is one of the main components in *Alisma orientale* (Sam.) Juz [3, 4]. Modern pharmacological investigations have shown that 24A is a multitarget compound that regulates the processes of inflammation, oxidative stress, autophagy, and apoptosis. Wu et al. reported that 24A suppresses oxidative stress and stimulates autophagy through the Adenosine 5′-monophosphate (AMP)-activated protein kinase (AMPK)/mammalian target of rapamycin (mTOR) pathway in mice with nonalcoholic steatohepatitis [5]. Another in vitro study indicated that 24A effectively reduces the levels of inflammatory factors, such as TNF-α, IL-1β, IL-6, and IL-8 in HepG2 cells [6]. In addition, as shown in our previous study, 24A exerts an antiapoptotic effect and protects the tight junctions of brain microvascular endothelial cells from damage caused by oxygen and glucose deprivation [7, 8]. However, the potential neuroprotective role of 24A in ischaemic stroke remains to be elucidated.

Phosphatidylinositol 3-kinase (PI3K) induces the phosphorylation of protein kinase B (AKT) in response to extracellular signals to regulate cell survival, growth, and angiogenesis. Previous studies have reported a pivotal role for PI3K/AKT signalling in defending against various insults that damage neurons [9]. In an oxygen–glucose deprivation and reperfusion (OGD/R) model in vitro, PI3K/AKT expression is inhibited, indicating that I/R injury inhibits the PI3K/AKT pathway [10]. Neuroinflammation and cell apoptosis are inhibited by activating PI3K/AKT signalling after cerebral ischaemia [11]. Overall, these results indicate that the upregulation of the PI3K/AKT pathway exerts a strong neuroprotective effect by reducing neuroinflammation and apoptosis caused by brain I/R injury. In this study, we evaluated the anti-neuroinflammatory and anti-apoptotic effects of 24A on mice with global cerebral ischaemia–reperfusion (GCI/R) injury and explored its potential mechanism. We verified our hypothesis that the PI3K/AKT signalling pathway was involved in the anti-inflammatory and antiapoptotic effects of 24A by administering the PI3K inhibitor LY294002 in rescue experiments. The results of this study provide a scientific basis for the clinical application of 24A as a treatment for ischaemic brain diseases.

## Materials and methods

### Animals

C57BL/6J mice (male, weighing 25–30 g, 12 weeks) were purchased from GemPharmatech (Jiangsu, China). Mice were housed in standard cages in the Animal Experimental Centre of Fujian University of Traditional Chinese Medicine and allowed to eat and drink freely. The environmental facility license no. was SYXK (Min) 2019-0007. All experimental procedures were approved by the Ethics Committee of Fujian University of Traditional Chinese Medicine and were performed in strict accordance with the animal care and use guidelines of the National Institutes of Health.

### Surgical procedures

The two-vessel occlusion (2-VO) method was performed to establish a model of global cerebral ischaemia (GCI), as previously described [12]. Mice were fasted for 24 h before surgery. Then, the mice were initially anesthetized with 1.5% thiopental sodium by intraperitoneal injection and maintained with oxygen and isoflurane. The common carotid artery (CCA) was gently isolated from vagus. Next, the 4.0 silk suture was performed to occlude both common carotid arteries (CCAs) for 20 min and induce transient global cerebral ischaemia–reperfusion injury (GCI/R). Blood flow was restored after 20 min to induce reperfusion.

### Drug management and experimental subgroups

24A was suspended in normal saline in powder form. PI3K inhibitor LY294002 was dissolved in dimethyl sulfoxide (DMSO) and diluted with normal saline when used. The final content of DMSO did not exceed 0.1%. Mice were randomly divided into four groups (*n* = 12 mice per group): sham group (only the external carotid artery and vagus nerve were separated without drug intervention), GCI/R group (reperfusion was performed after 20 min of global cerebral ischaemia, GCI/R + 24A group (24 h after reperfusion, 30 mg/kg 24A was administered by gavage once a day for 7 consecutive days), and GCI/R + 24A + LY group (24 h after reperfusion, 30 mg/kg 24A was administered by gavage for 7 days, and PI3K inhibitor LY294002 (10 mg/kg once a day) was injected intraperitoneally in the first 3 days).

### Neurological deficit score

The modified neurological deficit score (mNSS) was used to evaluate neurological deficits, including motor, sensory, reflex and balance ability tasks. Neurological defect scores were determined on the first, third, fifth and seventh days after global cerebral ischaemia–reperfusion. Scores ranged from 0 to a maximum of 18 points.

### Morris water maze

The Morris water maze (MWM) is designed to test the spatial memory and long-term memory of mice, which includes spatial exploration experiments and directional navigation experiments. In the spatial exploration experiment, mice were placed in the water within the first quadrant daily, and the time to find the platform within 90 s was recorded as the escape latency of the mice in the first quadrant. If the mice were unable to find the platform within 90 s, then the escape latency was recorded as 90 s, and mice were guided to the platform and allowed to stay on the platform for 15 s to learn its location. Each animal performed 4 trials per day and swam to an escape platform. The average escape latency in the four quadrants was recorded as the escape latency of the day. These steps were repeated daily for 4 days. The directional navigation experiment was conducted on the fifth day after the platform was removed. The mice were placed in the pool on the opposite side of the original platform quadrant. The number of times the mice passed the original platform position and the time spent in the target quadrant within 90 s were recorded.

### Novel object recognition test

The recognition memory of each mouse was evaluated using the novel object recognition test (NORT). 24 h before the test, the mice were allowed to explore an empty arena for 5 min. 24 h after habituation, the mice were exposed to the familiar arena containing two identical objects for 5 min. 24 h later, the mice were placed individually in the arena with one familiar object and one novel object. The time mice spent exploring novel objects and familiar objects was recorded for 5 min, and the discrimination index and time ratio were calculated using the formula: discrimination index = time spent exploring the novel object/total time spent exploring the familiar and novel objects, time ratio = time spent exploring the novel object/total time spent exploring the familiar and novel objects. The total exploration time and times spent exploring both objects were recorded at the same time.

### Magnetic resonance imaging

Mice were anaesthetized with a mixture of 1.5–2% isoflurane and oxygen. The specific parameters of T2-weighted image (T2W1) were echo time (TE) = 35 ms, repetition time (TR) = 4200 ms, average = 4, slice thickness = 0.5 mm, and field of view = 20 mm × 20 mm. The specific parameters of echo planer imaging (EPI) were TE = 25 ms, TR = 12,000 ms, average = 2, slice thickness = 0.5 mm, and field of view = 20 mm × 20 mm. Finally, a magnetic resonance spectroscopy (MRS) scan was performed, and T2 images of the bilateral hippocampus were recorded. The cortex was selected as the region of interest on the transverse, coronal, and sagittal planes of the right hemisphere, with a size of 1 mm × 1 mm × 1 mm. Specific parameters of MRS were TR = 1500 ms, TE = 144 ms, and number of averages = 256. The postprocessing of images and related data were analysed using the workstation TOPSPIN (V3.1, Bruker Biospin, Germany) of the MRI instrument. Creatine (Cr) was used as an internal reference. The spectrum peak positions were as follows: Cr, approximately 3.05 ppm; N-acetylaspartate (NAA), approximately 2.02 ppm; Complex of glutamate and glutamine (Glx), approximately 2.2–2.4 ppm; Myoinositol (MI), approximately 3.56 ppm; Taurine, approximately 3.4 ppm; and Choline (CHO), approximately 3.2 ppm. γ-aminobutyric acid (GABA) approximately 3.02 ppm.

Three-dimensional (3D) time of flight (TOF) magnetic resonance angiography (MRA) method, a non-invasive MRI-based flow imaging technique, was used to observe detailed images of cerebral blood vessels of GCI/R mice. Specific parameters of 3D TOF were TR = 15 ms, TE = 2.7 ms, Field of view = 30 × 30 × 24 mm, averages = 1, slices = 1, slice thickness = 24 mm, time = 5 min 5 s 280 ms.

### Immunohistochemistry

After rodents were anaesthetized, 0.9% normal saline was perfused through the heart followed by 4% precooled paraformaldehyde, and then the brain was collected. The brain was fixed with 4% paraformaldehyde, embedded in paraffin, and cut into 4 μm paraffin sections. The immunohistochemical process was performed according to the instructions of the immunohistochemistry (IHC) Kit (KIT-9720, MXB, Biotechnologies, Fujian, China). After dewaxing and rehydration, the sections were immersed in a beaker containing antigen repair solution, heated for 20 min, cooled naturally for 2 h, and washed with phosphate buffered saline (PBS) 3 times. After an incubation for 15 min with 3% H_2_O_2_ to eliminate the effects of endogenous peroxidases, the slices were then washed with PBS 3 times. Next, the sections were incubated with blocking buffer at room temperature for 1 h and then incubated with a primary antibody against Iba1 (1:200, Proteintech, Cat No.: 10904-1-AP), GFAP (1:1000, Proteintech, Cat No.:16825-1-AP), or NeuN (1:2000, Proteintech, Cat No.:26975-1-AP 1:2000) at 4 °C overnight and subsequently incubated with a secondary antibody. The sections were incubated with DAB-0031 (MXB, biotechnology, Fujian, China) for 1–10 min until a brown colour formed. Images of GFAP, Iba1, and NeuN staining in the hippocampus and cortex were captured using an optical microscope (Nikon, Model Eclipse Ci-L, 718345, Japan) and analysed with an image analysis system (ImageJ, version 6.0; Motic China Group Co., Ltd., Xiamen, China).

### Golgi staining

According to the instructions of the Rapid GolgiStain™ Kit (PK401, FD NeuroTechnologies, Columbia, USA), staining Solutions A and B were mixed 24 h in advance. After the mice were anaesthetized, their brains were removed, placed the mixed solution, and stored in the dark at room temperature for 2 weeks. Two weeks later, brain tissues were transferred to Solution C and stored at room temperature in the dark for 1 week. The brains were sliced into coronal section (100 μm) using a microtome with a vibrating blade (Leica VT1000 S, Leica, Nussloch, Germany), and sections were mounted on gelatine-coated microscope slides with Solution C. Each brain was cut into 5 slices and dried in the dark at room temperature. The staining solution was prepared by mixing an equal volume of Solutions D and E. After staining, slices were dehydrated, rendered transparent and sealed, and photographed under an optical microscope to observe the morphology.

### Western blot

Mouse brain tissues were collected, and then the protein was extracted. The protein concentration was determined using the BCA protein assay. Proteins were electrophoretically separated on 10% sodium dodecyl sulfate–polyacrylamide-gel electrophoresis (SDS–PAGE) gels (Bio-Rad Laboratories, Inc. Hercules, CA, USA) and transferred to polyvinylidene fluoride (PVDF) membranes. The PVDF membranes were blocked with 5% milk for 2 h, incubated with primary antibodies against IL-1β (1:1000, Abcam, Cat# Ab9722), PI3K (1:1000, Cell Signaling Technology, Cat# 4257), AKT (1:1000, Cell Signaling Technology, Cat# 4691), p-PI3K (1:1000, Cell Signaling Technology, Cat# 17366), p-AKT (1:1000, Cell Signaling Technology, Cat# 4060), TNF-α (1:1000, Cell Signaling Technology, Cat# 11948), Bcl-2 (1:1000, Proteintech, Cat# 26593-1-AP), Bax (1:2000, Proteintech, Cat# 50599-2-lg), cle-Caspase-3 (1:1000, Proteintech, Cat# 19677-1-AP), or GAPDH (1:7000, Proteintech, Cat# 60004-1-lg) at 4 °C for 12 h, and then incubated with a secondary antibody (1:7000) for 2 h. The membrane was detected with enhanced chemiluminescence kit (Meilunbio, MA0186). The protein bands were imaged with the Bio-Image Analysis system (Bio-Rad Laboratories, Inc.).

### Statistical analysis

Data are presented as the means ± standard error of the mean (SEM) and were analysed using one-way or two-way repeated measures analysis of variance (ANOVA) with IBM SPSS Statistics statistical software (IBM SPSS Statistics for Windows, Version 23.0. Armonk, NY, USA). After ANOVA, pairwise comparisons were analysed using the least significant difference test (LSD-t). A *p* value < 0.05 was defined as statistically significant.

## Results

### 24A ameliorated the neurological deficits of GCI/R mice

**Fig. 1:**
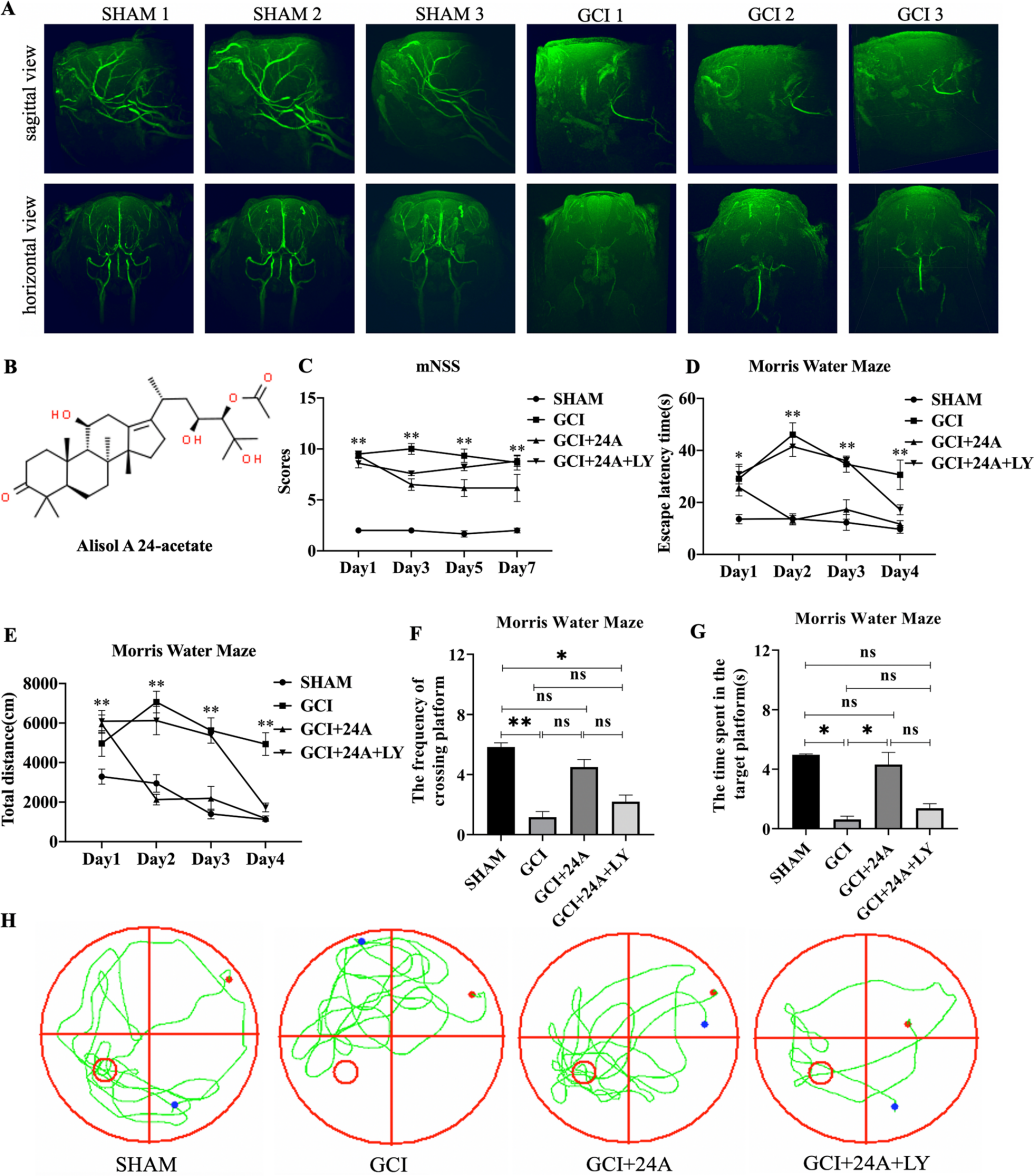
Treatment with 24A improved the neurological deficits of GCI/R mice. **A** Cerebral blood vessel of mice detected by three-dimensional time of flight magnetic resonance angiograph method. **B** The molecular structure of 24A. **C** The neurological deficit scores evaluated on the first, third, fifth and seventh days (*n* = 12, repeated measures ANOVA, *p* < 0.001). **D**, **E** Time (*n* = 12, repeated measures ANOVA, *p* < 0.001) and distance (*n* = 12, repeated measures ANOVA, *p* < 0.001) of the mice travelled to find the platform in the spatial exploration experiment of the MWM. **F**, **G** Frequency of the mice crossing over the target platform location (*n* = 12, one-way ANOVA, *p* = 0.001) and the time spent in the target platform quadrant (*n* = 12, one-way ANOVA, *p* = 0.040) in the directional navigation experiment of the MWM. H Representative trajectories in the directional navigation experiment of the MWM. (**p < 0.01, *p < 0.05, ns indicates no significant differences)

The molecular structure of 24A is shown in Fig. 1B. We used three-dimensional (3D) time of flight (TOF) magnetic resonance angiography (MRA) method to detect the cerebral blood vessels of mice. The images (Fig. 1A) showed that two-vessel occlusion surgery we performed successfully occluded both common carotid arteries. We scored neurological deficits on the first, third, fifth, and seventh days after ischaemia–reperfusion injury. According to the line chart (Fig. 1C), the neurological deficit scores of all groups displayed a downwards trend. The score of the GCI/R group was significantly higher than that of the sham group. After the 24A intervention, the score decreased, while the use of the PI3K inhibitor reversed the effect of 24A. Based on these results, 24A ameliorated the neurological deficits, and the effect was reversed by the PI3K inhibitor LY294002.

### 24A improved GCI/R-induced spatial memory and learning deficits

The Morris water maze was used to test the learning and memory abilities of the animals. Escape latency and the total distance travelled represent the time and distance required for mice to find the target platform. A shorter escape latency and total distance represent better spatial memory and learning abilities. The escape latency and total distance showed a decreasing trend in the spatial exploration experiment (Fig. 1D, E). Compared with the sham group, GCI/R mice required a longer time and exhibited a longer path length when searching for the platform. After the 24A intervention, the escape latency and total distance travelled decreased significantly compared with the GCI/R group, indicating that 24A significantly improved the spatial memory and learning abilities of mice. The escape latency of the PI3K inhibitor group was longer than that of the sham group and 24A intervention group. Although the difference was not significant, it still indicated that the addition of the inhibitor prevented the improvements in the spatial memory and learning abilities of mice to some extent. After the spatial exploration experiment, the frequency at which mice crossed the target platform location and the time spent in the platform location were tested. Compared with the sham group, the numbers of platform crossings (Fig. 1F) were significantly reduced in the GCI/R group (*p* < 0.01) and inhibitor group (*p* < 0.05). After the 24A intervention, the number of platform crossings increased, although the difference was not significant (*p* > 0.05). Compared with the GCI/R group, the sham group and 24A intervention group spent more in the platform quadrant (Fig. 1G) (*p* < 0.05). Mice in the sham group tended to swim to the zone, where the platform was previously located, while mice in the GCI/R group did not exhibit this preference. The 24A treatment increased the proportion of distance travelled and time spent in the platform quadrant, while the effect was reversed by the PI3K inhibitor LY294002 (Fig. 1H).

### 24A improved the ability of GCI/R mice to explore novel objects

**Fig. 2:**
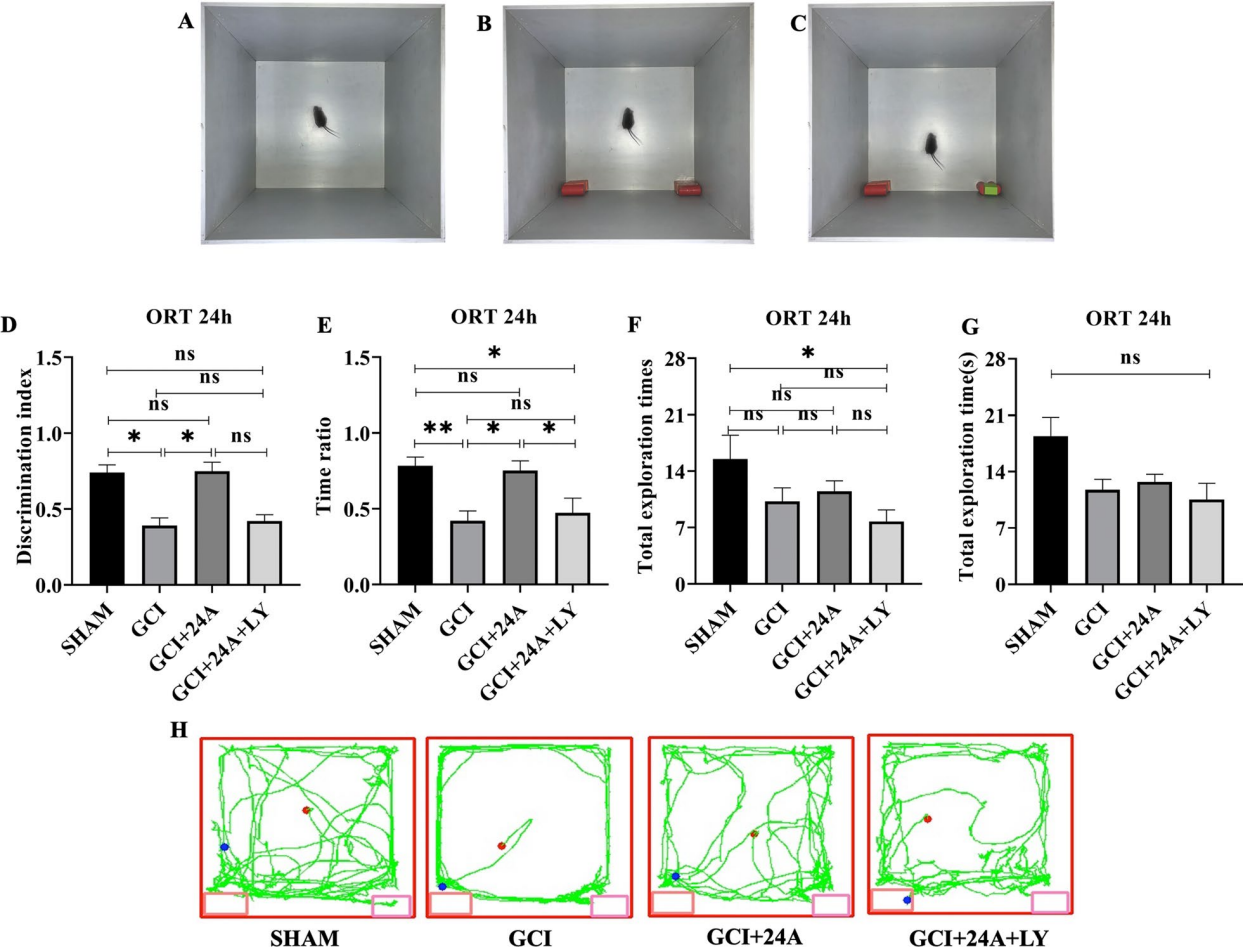
Treatment with 24A improved the ability of GCI/R mice to explore novel objects. **A**–**C** Schematic diagrams of the novel object recognition test. **D**, **E** Discrimination index (*n* = 12, one-way ANOVA, p = 0.002) and time ratio (*n* = 12, one-way ANOVA, *p* = 0.010) in the ORT at 24 h. (Discrimination index = time spent exploring the novel object/total time spent exploring the familiar and novel objects), (time ratio = time spent exploring the novel object/total time spent exploring familiar and novel objects). **F**, **G** Total exploration frequencies (*n* = 12, one-way ANOVA, *p* = 0.159) and total exploration time (*n* = 12, one-way ANOVA, *p* = 0.076) in the ORT at 24 h. **H** Representative trajectory diagram in the ORT at 24 h. (**p < 0.01, *p < 0.05, ns indicates no significant differences)

NORT are based on the principle that animals have a tendency to explore novel objects (Fig. 2A–C). The discrimination index and time ratio represent the frequency and time mice spend exploring a novel object relative to that of the familiar object. As shown in Fig. 2D, the GCI/R group showed a lower discrimination index than the sham group at 24 h in the NORT (*p* < 0.05). Treatment with 24A significantly increased the discrimination index (*p* < 0.05), while the PI3K inhibitor decreased it, indicating that 24A improved the ability of mice to explore and distinguish novel objects and confirmed its effect on improving long-term memory. The time ratio (Fig. 2E) is the time animals spend exploring the novel object relative to the total exploration time. The ratio in the GCI/R group was less than that in the sham group at 24 h in the NORT (*p* < 0.01). After the 24A intervention, the time ratio increased (*p* < 0.05), and the inhibitor reduced this value (*p* < 0.05). Therefore, GCI/R mice spent more time exploring novel objects after the 24A intervention, which confirmed that 24A improved the ability of mice to explore novel objects. The exploration trajectory of mice was consistent with the phenomenon described above (Fig. 2H). However, no significant difference in total exploration times were observed between the 4 groups (Fig. 2F, G).

### 24A improved the changes in substance metabolism in various brain regions of mice with I/R injury

**Fig. 3:**
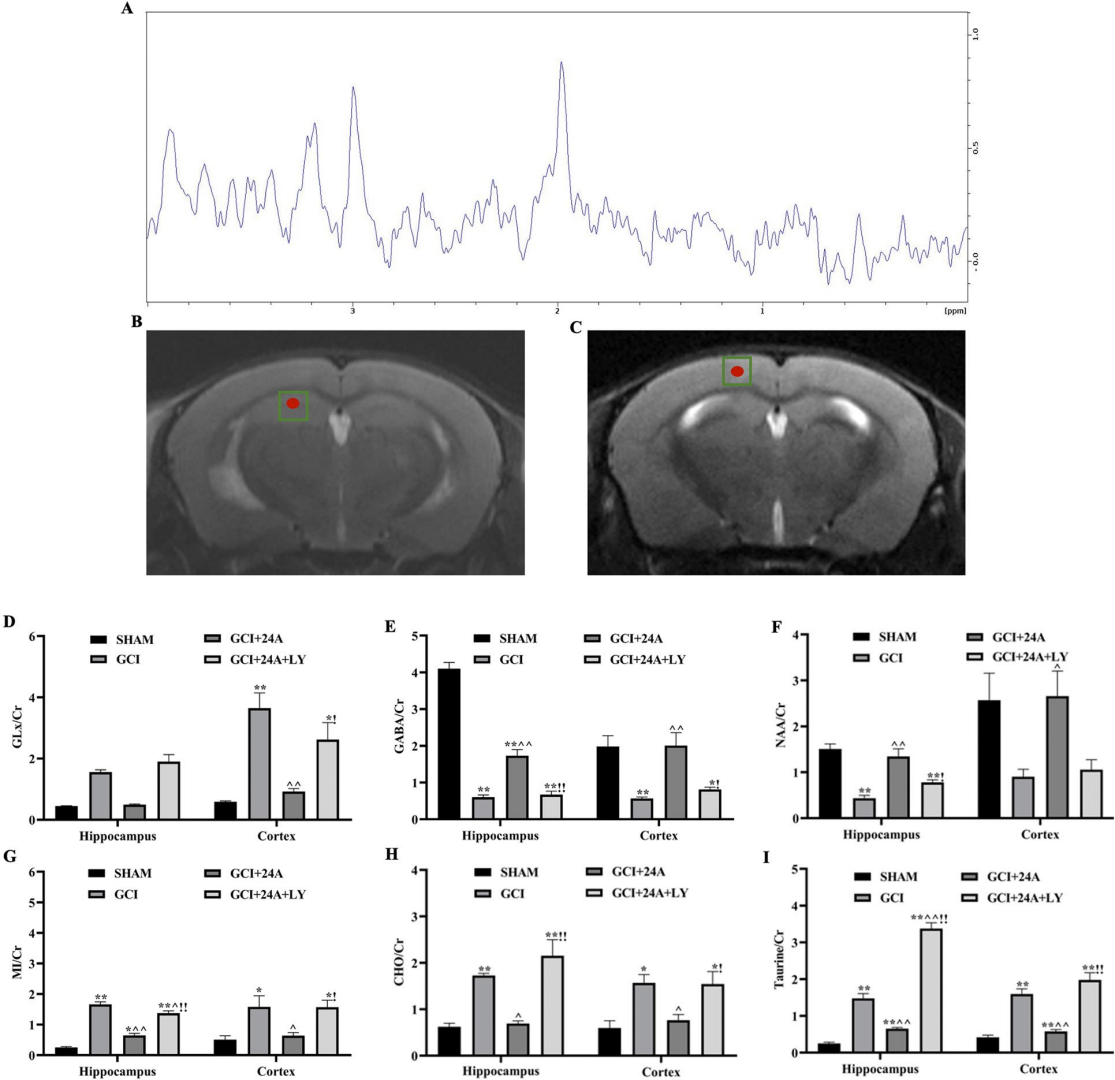
Treatment with 24A improved metabolism in different brain regions of GCI/R mice. Substance metabolism in the hippocampus and cortex was detected using MRS. **A** Typical spectra recorded from the hippocampus of mice. **B**, **C** Region of interest (ROI) in the mice hippocampus and cortex. **D**–**I** Changes in the levels of the metabolites Glx (*n* = 6, Kruskal–Wallis test/one-way ANOVA, *p*_hippocampus_ = 0.027, *p*_cortex_ = 0.005), GABA (*n* = 6, one-way ANOVA, *p*_hippocampus_ < 0.001, *p*_cortex_ = 0.012), NAA (*n* = 6, one-way ANOVA, *p*_hippocampus_ = 0.002, *p*_cortex_ = 0.079), MI (*n* = 6, one-way ANOVA, *p*_hippocampus_ < 0.001, *p*_cortex_ = 0.041), CHO (*n* = 6, one-way ANOVA, *p*_hippocampus_ = 0.003, *p*_cortex_ = 0.033) and Taurine (*n* = 6, one-way ANOVA, *p*_hippocampus_ < 0.001, *p*_cortex_ < 0.001) in the hippocampus and cortex. (*p < 0.05 and **p < 0.01 compared with the sham group; ^*p* < 0.05 and ^^*p* < 0.01 compared with the GCI group; !*p* < 0.05 and ^!!^*p* < 0.01 compared with the 24A intervention group)

In our study, MRS was used to detect changes in neurometabolites in the hippocampus and cortex (Fig. 3A–C). Because creatine (Cr) is present at relatively constant and uniform levels, it is usually used as an internal reference for measuring the levels of other metabolites. In our study, Glx/Cr (Fig. 3D) levels in the cortex of the GCI/R group increased, while 24A treatment reduced Glx/Cr levels in the cortex (*p* < 0.01). The GCI/R group showed a decrease in GABA/Cr levels (Fig. 3E) in the hippocampus (*p* < 0.01) and cortex (*p* < 0.01) but an increase after the addition of 24A (*p* < 0.01). In our study, significantly decreased NAA/Cr levels (Fig. 3F) were detected in the hippocampus of the GCI/R group (*p* < 0.05), while 24A increased the levels of this metabolite (*p* < 0.01), but the change in the cortex was not significant (*p* > 0.05). The MI/Cr ratio (Fig. 3G) increased in the hippocampus (*p* < 0.01) and cortex (*p* < 0.05) in the GCI/R group increased but decreased after the 24A intervention in the hippocampus (*p* < 0.01) and cortex (*p* < 0.05). The contents of CHO (Fig. 3H) increased in the hippocampus (*p* < 0.01) and cortex (*p* < 0.05) of the GCI/R group but decreased after treatment with 24A (*p* < 0.01, *p* < 0.05). I/R injury leads to an increase in Taurine levels in the hippocampus and cortex. Our study confirmed significantly higher Taurine levels (Fig. 3I) in the GCI/R group than in the sham group (*p* < 0.01), while the 24A intervention reduced Taurine levels (*p* < 0.01), and the administration of the PI3K inhibitor increased its levels (*p* < 0.01).

### 24A reduced GCI/R-induced microglial and astrocyte activation

**Fig. 4:**
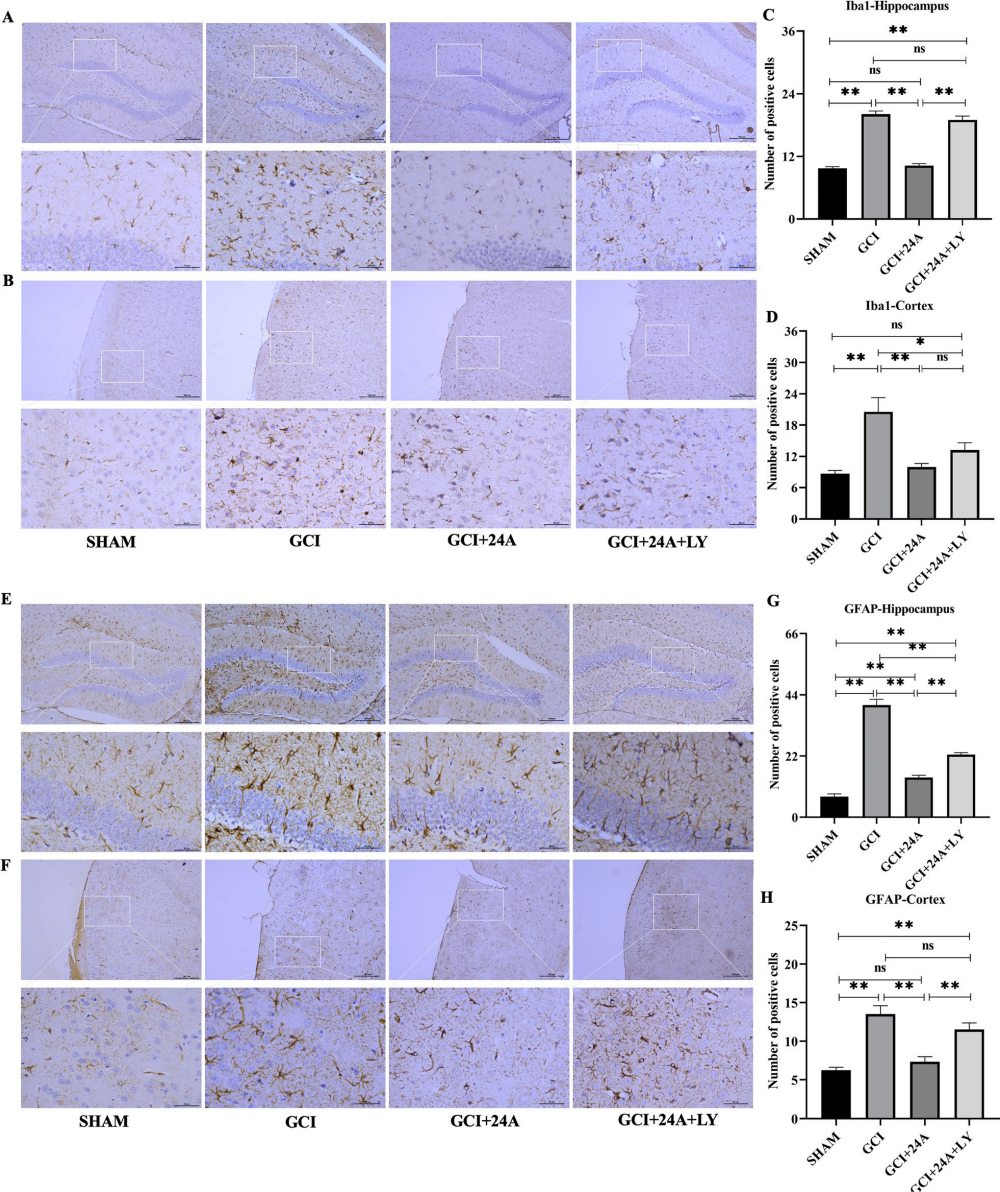
Treatment with 24A reduced GCI/R-induced microglial and astrocyte activation. **A**, **B** Immunohistochemical staining for Iba1 in the hippocampus and cortex (10 ×, 40 × magnification). **C**, **D** Quantitative analysis of microglia in the hippocampus (*n* = 3, one-way ANOVA, *p* < 0.001) and cortex (*n* = 3, one-way ANOVA, *p* = 0.011). **E**, **F** Immunohistochemical staining for GFAP in the hippocampus and cortex (10 ×, 40 × magnification). G, H Quantitative analysis of astrocytes in the hippocampus (*n* = 3, one-way ANOVA, *p* < 0.001) and cortex (*n* = 3, one-way ANOVA, *p* < 0.001). (**p < 0.01, *p < 0.05, ns indicates no significant differences)

Ionized calcium binding adaptor molecule 1 (Iba1) is a positive marker of microglia, and glial fibrillary acidic protein (GFAP) is a positive marker of astrocytes. In the GCI/R group, the number of Iba1 + and GFAP + cells in the hippocampus (Fig. 4A, E) and cortex (Fig. 4B, F) increased, and the cell body became larger, indicating that microglia and astrocytes were overactivated after GCI/R (*p* < 0.01). The numbers of microglia and astrocytes in the 24A group were markedly decreased (*p* < 0.01). Notably, 24A inhibited microglial and astrocyte overactivation in the hippocampus and cortex. However, this effect was reversed by the PI3K inhibitor (*p* < 0.01), although Iba1 levels in the cortex were not significantly different. The numbers of microglia and astrocytes in the hippocampus and cortex were calculated using ImageJ software (Fig. 4C, D, G, H).

### 24A attenuated GCI/R-induced inflammation and apoptosis

**Fig. 5:**
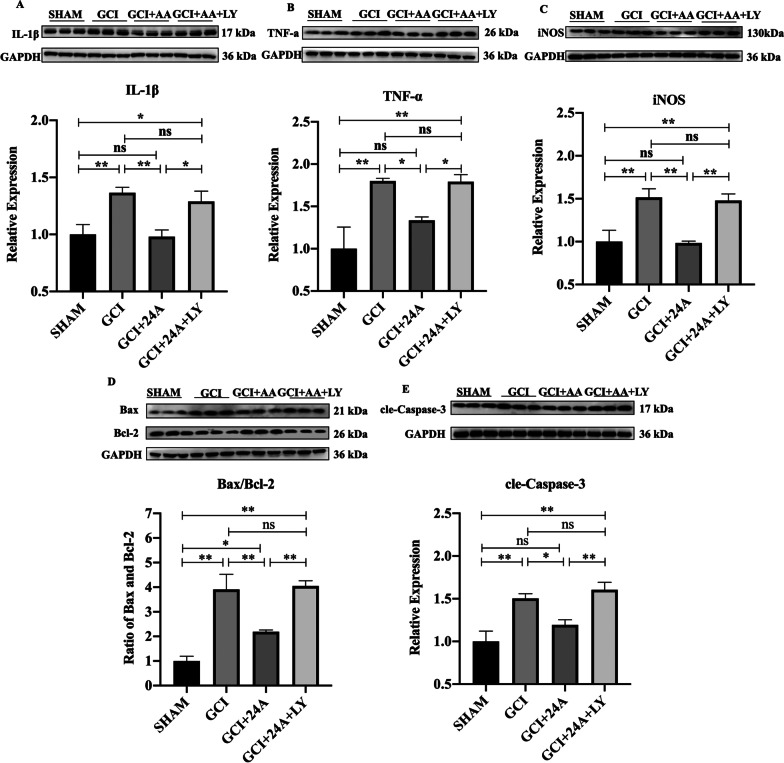
Treatment with 24A attenuated GCI/R-induced inflammation and apoptosis. **A**–**E** Representative western blot images and quantitative analysis of IL-1β (*n* = 3, one-way ANOVA, *p* = 0.011), TNF-α (*n* = 3, one-way ANOVA, *p* = 0.008), iNOS (*n* = 3, one-way ANOVA, *p* = 0.004), Bax/Bcl-2 (*n* = 3, one-way ANOVA, *p* = 0.001), and cle-Caspase-3 (*n* = 3, one-way ANOVA, *p* = 0.003) levels. (**p < 0.01, *p < 0.05, ns indicates no significant differences)

We detected the expression of proteins related to inflammation and apoptosis using western blot to better understand the anti-inflammatory and antiapoptotic mechanisms of 24A. IL-1β, TNF-α and iNOS were expressed at higher levels in the GCI/R group than in the sham group (*p* < 0.01), and the 24A intervention markedly attenuated the increased expression of IL-1β (*p* < 0.01), TNF-α (*p* < 0.05) and iNOS (*p* < 0.01) but these changes were reversed by the PI3K inhibitor LY294002 (Fig. 5A–C). Thus, 24A reduced inflammation induced by ischaemia–reperfusion. The levels of Bax and cleaved Caspase-3 were significantly increased after GCI/R injury, while Bcl-2 levels were decreased (*p* < 0.01). Treatment with 24A significantly reduced the ratio of Bax to Bcl-2 (*p* < 0.01) and the expression of cleaved Caspase-3 (*p* < 0.05). However, the situation was reversed by the PI3K inhibitor LY294002 (Fig. 5D, E). Notably, 24A inhibited inflammation and apoptosis after GCI/R by regulating the levels of proinflammatory factors and proapoptotic and antiapoptotic proteins. However, LY294002 reversed the effect of 24A.

### Therapeutic effect of 24A on GCI/R-induced apoptotic neuronal death

**Fig. 6:**
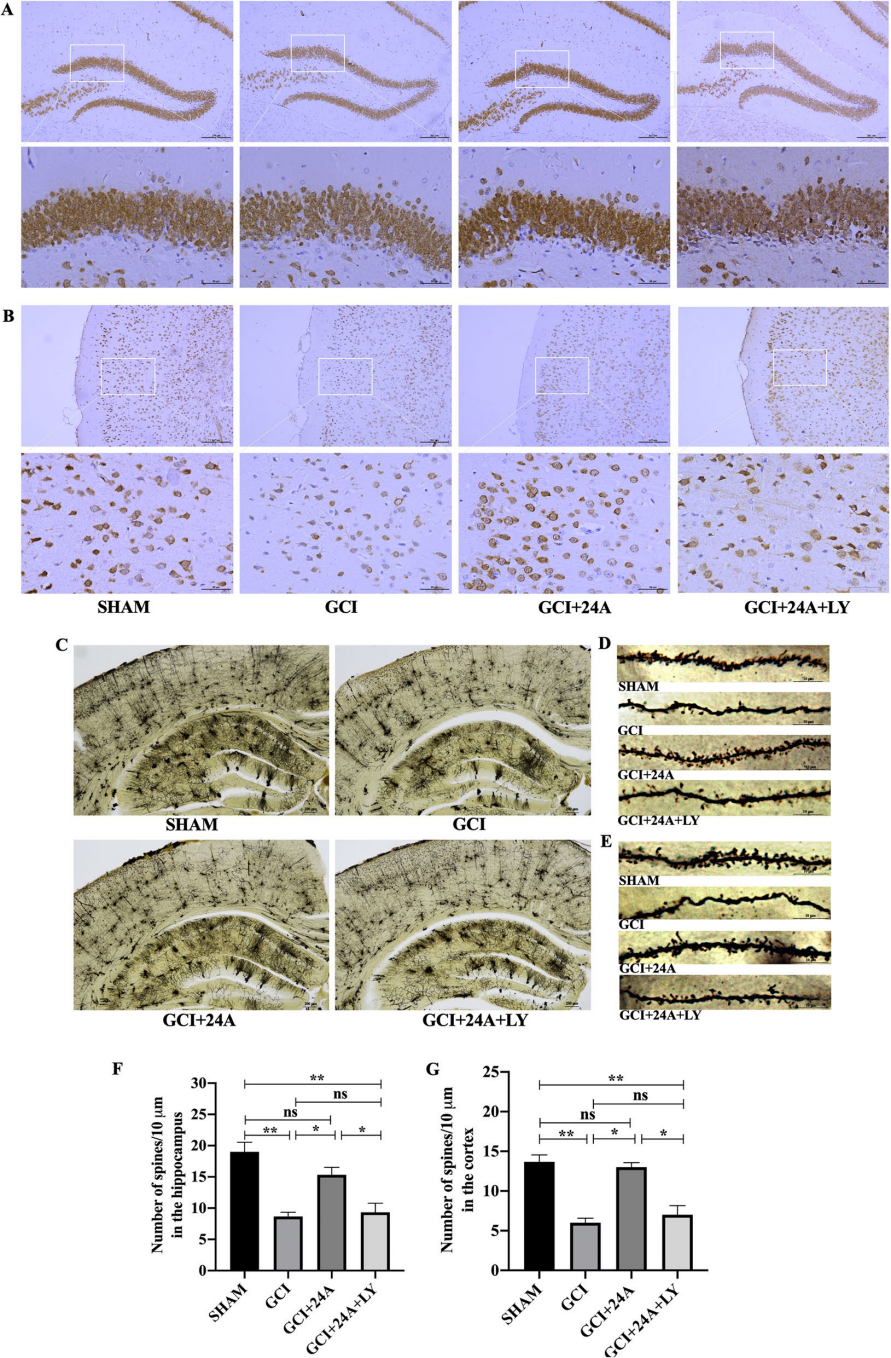
Therapeutic effect of 24A on GCI/R-induced apoptotic neuronal death. **A**, **B** Immunohistochemical staining of neurons in the hippocampus and cortex (10 ×, 40 × magnification). **C** The quantity and arrangement of neurons in the hippocampus and cortex were observed using Golgi staining (4 × magnification). **D**, **E** Density of dendritic spines on hippocampal and cortical neurons was observed using Golgi staining (100 × magnification). **F**, **G** Quantitative analysis of dendritic spine density of hippocampal (*n* = 3, one-way ANOVA, *p* = 0.001) and cortical neurons (*n* = 3, one-way ANOVA, *p* < 0.001) in each group. (**p < 0.01, *p < 0.05, ns indicates no significant differences)

More obvious morphological changes in neurons, such as nuclear shrinkage, chromatin deepening, irregular cell morphology and blurred edges, were observed in the hippocampus and cortex. The addition of 24A attenuated the destruction of neurons, and the morphology was relatively regular. However, the addition of the PI3K inhibitor reversed this change (Fig. 6A, B).

### Therapeutic effect of 24A on GCI/R-induced damage to hippocampal and cortical neurons and dendritic spines

Golgi staining was performed to observe the quantity and arrangement of hippocampal and cortical neurons in each group and to further investigate the destruction of the neuronal structure. As shown in Fig. 6C, the number of hippocampal neurons was significantly decreased and the remaining cells were arranged irregularly in the GCI/R group compared with the sham group. After the 24A intervention, the number of hippocampal neurons increased and cells were arranged neatly and regularly, while the administration of the PI3K inhibitor weakened the effect of 24A (Fig. 6C). Furthermore, we found that the dendritic spines on hippocampal neurons in the CA1 region and cortical neurons (Fig. 6D–G) of the sham group were dense. While in the GCI/R group, the dendritic spines were lost and atrophied, and the number of dendritic spines was decreased compared with the sham group (*p* < 0.01). A reduction in the dendritic spine density had been shown to be associated mainly with brain dysfunctional. Although some dendritic spines in the 24A intervention group were lost, the number of dendritic spines was higher than that in the GCI/R group (*p* < 0.05). The PI3K inhibitor decreased the number of dendritic spines (*p* < 0.05).

### 24A activated PI3K/AKT signalling pathway after GCI/R

**Fig. 7:**
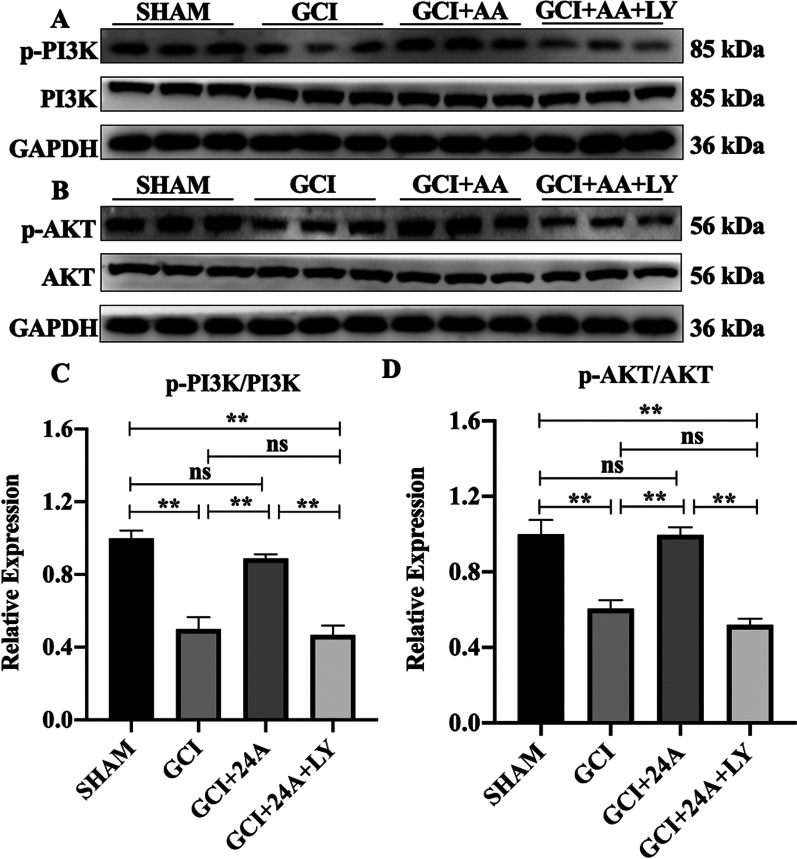
24A activated the PI3K/AKT pathway in the GCI/R model. **A**, **B** Levels of p-PI3K, PI3K, p-AKT, and AKT were detected using western blotting, with GAPDH serving as a loading control. **C**, **D** Ratio of p-PI3K to PI3K (*n* = 3, one-way ANOVA, *p* < 0.001) and p-AKT to AKT (*n* = 3, one-way ANOVA, *p* < 0.001) among different groups was analysed. (**p < 0.01, *p < 0.05, ns indicates no significant differences among each group)

We confirmed whether the PI3K/AKT signalling pathway was involved in the protective effect of 24A by first examining the changes in PI3K/AKT levels after ischaemia–reperfusion and the 24A intervention using western blot. Total PI3K and AKT levels were consistent among the four groups. Treatment with 24A increased PI3K phosphorylation compared with that in the GCI/R group, while LY294002 inhibited PI3K phosphorylation (Fig. 7A, B). Compared with the sham group, p-PI3K/PI3K and p-AKT/AKT levels were decreased after GCI/R (*p* < 0.01) and increased after the 24A intervention (*p* < 0.01), while the PI3K inhibitor reversed the effect of 24A (*p* < 0.01, Fig. 7C, D). PI3K is upstream of AKT, and the PI3K/AKT pathway is related to cell survival and growth, which may play an important role in attenuating cerebral ischaemia.

## Discussion

Ischaemic stroke is the primary cause of death and long-term disability in elderly individuals. Ischaemia reperfusion (I/R) is a complex pathological process accompanied by ischaemic stroke that exacerbates brain damage, including reactive oxygen species (ROS) outburst, inflammatory mediator overproduction, leukocyte infiltration, increased Ca^2+^ influx, and calcium overload, all of which mediate the induction of apoptosis [13]. These factors are considered to play important roles in neuronal injury after ischaemic injury [14]. Based on the pathogenesis of I/R, many neuroprotective drugs have been designed to protect the brain from reperfusion injury or to inhibit the pathological process. However, few effective treatments are currently available for ischaemic stroke. The present study confirmed that alisol A 24-acetate (24A), one of the main active triterpenes of Rhizoma Alismatis [4], significantly attenuated global cerebral ischaemia/reperfusion (GCI/R)-induced brain injury.

Studies have shown that microglia exert a proinflammatory and proapoptotic effect on the early stage of I/R [15] by regulating the immune response and affecting neuronal function. Microglia function in the early stage of ischaemia–reperfusion by undergoing morphological changes and secreting inflammatory factors [16]. Global cerebral ischaemia is usually followed by inflammation induced by astrocytes in the initial stage of inflammation. In the I/R group, the expression of GFAP, a characteristic marker of astrocytes, was upregulated, indicating that I/R injury may be accompanied by reactive astrogliosis. Many studies have revealed that activated astrocytes in ischaemic regions are potentially harmful, because they release chemokines and increase the levels of NOS and neurotoxic NO, thereby aggravating ischaemic brain diseases [17]. Furthermore, the hippocampus and cortex are vulnerable to microglial activation and reactive astrocytes in GCI/R [18]. In our study, the activation of microglia and astrocytes in the hippocampus and cortex of the GCI/R group increased, and the morphology changed significantly, which confirmed that the hippocampus and cortex may be damaged by GCI/R injury. The 24A intervention reduced the activation of microglia and astrocytes, indicating that 24A may effectively suppress inflammation in the process of GCI/R injury. IL-1β and TNF-α are the primary cytokines mediating inflammation in response to transient I/R, and the expression of iNOS is upregulated during I/R, which leads to ischaemic neural damage [19]. Furthermore, the expression of IL-1β, TNF-α and iNOS was suppressed by 24A treatment. In general, the inflammatory process continues during I/R and plays a vital role in the disease outcome. Our results are consistent with a study showing that TNF-α and IL-1β production were markedly inhibited by 24A [6]. Apparently, 24A-mediated downregulation of inflammatory factors suggests its potential as a great candidate in investigations of novel therapeutic targets for GCI/R injury.

The anti-inflammatory effect of 24A has been proven. Interestingly, the inhibition of neuroinflammation may reduce neuronal apoptosis [20]. Apoptosis is crucial for neuronal death after cerebral I/R injury, which is considered the prominent form of neuronal death occurring in the penumbra. The expression of apoptosis-related genes was upregulated in response to cerebral I/R injury. Bcl-2 is a key antiapoptotic factor, and Bax is a proapoptotic factor. Various reports suggest that Bcl-2 expression is decreased, while Bax expression is increased after reperfusion, and inhibition of Bcl-2 reduces apoptosis and neuroinflammation after I/R [21]. Caspases and the Bcl family play equally important roles in the regulation of apoptosis. Activation of caspase-3 is an important feature of apoptosis following I/R, and its levels increase in the hippocampus of individuals with I/R injury [22]. As shown in our previous study, 24A exerts an antiapoptotic effect on OGD-induced brain microvascular endothelial cells [8]. The in vitro experiments showed that 24A reduced the proportion of Bax/Bcl-2, indicating that 24A, an anti-neuroinflammatory drug, effectively ameliorated apoptosis. These findings are consistent with previous cellular studies [7, 8] showing that 24A exerts antiapoptotic effects by regulating the expression of Bax/Bcl-2 in vivo.

Ischaemic stroke contributes to the development of neurological deficits and cognitive decline. The modified neurological severity score (mNSS) and morris water maze (MWM) are often used as indicators to evaluate the neurological function of rodent models. We found that 24A significantly improved neurological function and spatial learning and memory following GCI/R injury. We observed the synaptic morphology in the hippocampus and cortex to further investigate the intrinsic mechanism underlying neuronal changes during GCI/R injury. Synaptic plasticity is the cellular physiological basis of learning and memory [23]. Dendritic spines are small processes of dendrites, and their dynamic changes are an important form of synaptic remodelling [24, 25]. Cerebral ischaemia affects the synaptic structure and the density of dendritic spines, which exhibit a continuously decreasing trend with the extension of the ischaemia time. In the present study, the dendritic branches and density of dendritic spines increased after the 24A intervention. Thus, 24A effectively promotes the remodelling of hippocampal and cortical dendritic spine structure and function to promote the recovery of neurological function after CI/R. The neuroprotective activity of 24A suggests that it represents a promising and effective drug for improving neurological function after I/R injury.

Magnetic resonance imaging (MRS) is a novel technique used to detect brain metabolites in vivo. Ischaemia induced neurochemical and metabolic changes in the brain, including alterations in creatine (Cr), lactic acid, N-acetylaspartate (NAA), γ-aminobutyric acid (GABA), myoinositol (MI) and glutamate (Glu), and glutamine levels. MI is a marker of glial hyperplasia, and its level is increased in some nervous system diseases. Choline (CHO) levels increased during cerebral ischaemia. Amino acid neurotransmitters are the most abundant neurotransmitters in the central nervous system. In pathophysiological processes, their concentrations will change substantially. During the early stage of cerebral ischaemia, neuronal damage may be induced by excitatory neurotoxicity due to the oversecretion of neurochemicals [26]. Glx is a complex of glutamate and glutamine, which is an excitatory neurotransmitter that participates in many important metabolic pathways. Under ischaemic conditions, its peak becomes larger. GABA is an inhibitory neurotransmitter that plays a key role in motor learning and mediates the recovery of brain injury, but its levels decrease after stroke. NAA, which is considered a predominantly neuronal marker, reflects the functional state of neurons. The inhibition of neuronal metabolism leads to a significant decrease in NAA/Cr levels during cerebral ischaemia, indicating the development of cognitive deficits. Our study revealed decreased NAA levels after I/R injury, consistent with existing studies [27]. A reduction in NAA levels is often regarded as an indicator of neuron loss [28]. Recent magnetic resonance imaging (MRI) studies have been conducted to explain the susceptibility of hippocampal neurons to metabolic stress and delayed neuronal death, such as glutamate excitotoxicity, changes in lipid metabolism and production of free radicals, energy metabolism and local blood flow dysfunction, and apoptosis induction [29]. This finding explains why ischaemia causes an increase in Glx levels and subsequently mediates neuronal death. Our experiments showed that 24A regulated the levels of these substances, which provides a research basis for its application in the protection against brain injury after cerebral I/R. Furthermore, the application of MRI provides a scientific basis for the monitoring of changes in cognitive function caused by altered neurotransmitter levels after cerebral ischaemia.

Inflammation and apoptosis caused by ischaemia–reperfusion injury exert a serious effect on neurons and ultimately damage cognitive function. In our study, the neuroprotective effect of 24A was weakened when the PI3K-AKT signalling pathway was inhibited in GCI/R mice. We verified that this protective effect was mediated via the PI3K/AKT pathway by detecting behavioural performance, MRS, inflammation and apoptosis-related factors. We found that 24A activated the PI3K/AKT signalling pathway. Recent reports have highlighted the critical role of the PI3K/AKT signalling pathway in promoting cell survival. Bax and Bcl-2 are downstream proteins in the PI3K/AKT signalling pathway that regulate the activation of caspases [30]. Phosphorylated AKT increases the expression of the antiapoptotic protein Bcl-2 and mediates the inhibition of proapoptotic proteins. Recently, we observed increased expression of PI3K and AKT following 24A treatment. Therefore, we speculate that 24A may modulate PI3K/AKT signalling. As expected, inhibition of the PI3K/AKT pathway attenuated the protective anti-inflammatory and antiapoptotic effects of 24A treatment. In addition, the neurological deficits were exacerbated, and the behavioural performance deteriorated following the administration of LY294002. This result further indicated that the PI3K/AKT signalling pathway is involved in the protective effect of 24A on GCI/R injury.

## Conclusions

Taken together, our study first found that 24A exerts anti-inflammatory and anti-apoptotic effects and improves neurological deficits via the PI3K/AKT signalling pathway in a GCI/R model, which provides new insights and inspiration for the treatment of cerebral ischaemia–reperfusion injury**.**

## Supplementary Information

**Additional file 1.** Monitoring of cerebral blood flow and detection of brain tissue ischemia in mice.

## Data Availability

The data sets used and/or analysed during the current study are available from the corresponding author upon reasonable request.

## Abbreviations

24A: Alisol A 24-acetate
AKT: Protein kinase B
CBF: Cerebral blood flow
CCA: Common carotid artery
CCAs: Common carotid arteries
CHO: Choline
CI/R: Cerebral ischaemia–reperfusion
Cr: Creatine
DMSO: Dimethyl sulfoxide
EPI: Echo planer imaging
GABA: γ-Aminobutyric acid
GCI/R: Global cerebral ischaemia–reperfusion
GFAP: Glial fibrillary acidic protein
Glu: Glutamate
Glx: Complex of glutamate and glutamine
I/R: Ischaemia–reperfusion
Iba1: Ionized calcium binding adaptor molecule 1
IHC: Immunohistochemistry
IL: Interleukin
iNOS: Inducible nitric oxide synthase
LY: PI3K inhibitor LY294002
MI: Myoinositol
mNSS: Modified neurological severity score
MRI: Magnetic resonance imaging
MRS: Magnetic resonance spectroscopy
MWM: Morris water maze
NAA: N-acetylaspartate
NORT: Novel object recognition test
OGD/R: Oxygen–glucose deprivation–reoxygenation
PI3K: Phosphatidylinositol 3-kinase
ROS: Reactive oxygen species
SDS–PAGE: Sodium dodecyl sulfate–polyacrylamide-gel electrophoresis
T2W1: T2-weighted image
TE: Echo time
TNF-α: Tumor necrosis
TR: Repetition time

## References

[1] Wang J, Bai T, Wang N, Li H, Guo X (2020). Neuroprotective potential of imatinib in global ischemia-reperfusion-induced cerebral injury: possible role of Janus-activated kinase 2/signal transducer and activator of transcription 3 and connexin 43. Korean J Physiol Pharmacol.

[2] Sahota P, Savitz SI (2011). Investigational therapies for ischemic stroke: neuroprotection and neurorecovery. Neurotherapeutics.

[3] Xue X, Chen T, Wei W, Zhou X, Lin Z, Chen L (2014). Effects of Alisma Decoction on lipid metabolism and inflammatory response are mediated through the activation of the LXRalpha pathway in macrophage-derived foam cells. Int J Mol Med.

[4] Chen JX, Li HY, Li TT, Fu WC, Du X, Liu CH (2020). Alisol A-24-acetate promotes glucose uptake via activation of AMPK in C2C12 myotubes. BMC Complement Med Ther.

[5] Wu C, Jing M, Yang L, Jin L, Ding Y, Lu J (2018). Alisol A 24-acetate ameliorates nonalcoholic steatohepatitis by inhibiting oxidative stress and stimulating autophagy through the AMPK/mTOR pathway. Chem Biol Interact.

[6] Zeng L, Tang W, Yin J, Feng L, Li Y, Yao X (2016). Alisol A 24-acetate prevents hepatic steatosis and metabolic disorders in HepG2 cells. Cell Physiol Biochem.

[7] Lu L, Lu T, Shen J, Lv X, Wei W, Wang H (2021). Alisol A 24-acetate protects against brain microvascular endothelial cells injury through inhibiting miR-92a-3p/tight junctions axis. Aging (Albany NY).

[8] Zhou Y, Wei W, Shen J, Lu L, Lu T, Wang H (2021). Alisol A 24-acetate protects oxygen-glucose deprivation-induced brain microvascular endothelial cells against apoptosis through miR-92a-3p inhibition by targeting the B-cell lymphoma-2 gene. Pharm Biol.

[9] Ju SM, Kang JG, Bae JS, Pae HO, Lyu YS, Jeon BH (2015). The flavonoid apigenin ameliorates cisplatin-induced nephrotoxicity through reduction of p53 activation and promotion of PI3K/Akt pathway in human renal proximal tubular epithelial cells. Evid Based Complement Alternat Med..

[10] Zhao M, Hou S, Feng L, Shen P, Nan D, Zhang Y (2020). Vinpocetine protects against cerebral ischemia-reperfusion injury by targeting astrocytic Connexin43 via the PI3K/AKT signaling pathway. Front Neurosci.

[11] Wei Y, Hong H, Zhang X, Lai W, Wang Y, Chu K (2017). Salidroside inhibits inflammation through PI3K/Akt/HIF signaling after focal cerebral ischemia in rats. Inflammation.

[12] Zhu L, Wang L, Ju F, Ran Y, Wang C, Zhang S (2017). Transient global cerebral ischemia induces rapid and sustained reorganization of synaptic structures. J Cereb Blood Flow Metab.

[13] Ding Y, Kang J, Liu S, Xu Y, Shao B (2020). The protective effects of peroxisome proliferator-activated receptor gamma in cerebral ischemia-reperfusion injury. Front Neurol.

[14] Sun K, Fan J, Han J (2015). Ameliorating effects of traditional Chinese medicine preparation, Chinese materia medica and active compounds on ischemia/reperfusion-induced cerebral microcirculatory disturbances and neuron damage. Acta Pharm Sin B.

[15] Zeyen T, Noristani R, Habib S, Heinisch O, Slowik A, Huber M (2020). Microglial-specific depletion of TAK1 is neuroprotective in the acute phase after ischemic stroke. J Mol Med (Berl).

[16] Ma Y, Wang J, Wang Y, Yang GY (2017). The biphasic function of microglia in ischemic stroke. Prog Neurobiol.

[17] Galea E, Feinstein DL, Reis DJ (1992). Induction of calcium-independent nitric oxide synthase activity in primary rat glial cultures. Proc Natl Acad Sci U S A.

[18] Kho AR, Choi BY, Lee SH, Hong DK, Lee SH, Jeong JH (2018). Effects of protocatechuic acid (PCA) on global cerebral ischemia-induced hippocampal neuronal death. Int J Mol Sci.

[19] Arabian M, Aboutaleb N, Ajami M, Habibey R (2019). Interaction of mTOR and iNOS pathways in protection against ischemia/reperfusion injury. Iran J Pharm Res.

[20] Xie W, Zhu T, Dong X, Nan F, Meng X, Zhou P (2019). HMGB1-triggered inflammation inhibition of notoginseng leaf triterpenes against cerebral ischemia and reperfusion injury via MAPK and NF-kappaB signaling pathways. Biomolecules.

[21] Erfani S, Moghimi A, Aboutaleb N, Khaksari M (2019). Protective effects of nucleobinding-2 after cerebral ischemia via modulating Bcl-2/Bax ratio and reducing glial fibrillary acid protein expression. Basic Clin Neurosci.

[22] Liu G, Wang T, Wang T, Song J, Zhou Z (2013). Effects of apoptosis-related proteins caspase-3, Bax and Bcl-2 on cerebral ischemia rats. Biomed Rep.

[23] Matsuzaki M, Honkura N, Ellis-Davies GC, Kasai H (2004). Structural basis of long-term potentiation in single dendritic spines. Nature.

[24] Reza-Zaldivar EE, Hernandez-Sapiens MA, Minjarez B, Gomez-Pinedo U, Sanchez-Gonzalez VJ, Marquez-Aguirre AL (2020). Dendritic spine and synaptic plasticity in Alzheimer’s disease: a focus on MicroRNA. Front Cell Dev Biol.

[25] Liu W, Wu J, Huang J, Zhuo P, Lin Y, Wang L (2017). Electroacupuncture regulates hippocampal synaptic plasticity via miR-134-mediated LIMK1 function in rats with ischemic stroke. Neural Plast.

[26] He J, Zhao C, Liu W, Huang J, Liang S, Chen L (2018). Neurochemical changes in the hippocampus and prefrontal cortex associated with electroacupuncture for learning and memory impairment. Int J Mol Med.

[27] Kovalska M, Hnilicova P, Kalenska D, Tomascova A, Adamkov M, Lehotsky J (2020). Effect of methionine diet on time-related metabolic and histopathological changes of rat hippocampus in the model of global brain ischemia. Biomolecules.

[28] Berthet C, Xin L, Buscemi L, Benakis C, Gruetter R, Hirt L (2014). Non-invasive diagnostic biomarkers for estimating the onset time of permanent cerebral ischemia. J Cereb Blood Flow Metab.

[29] Wang W, Liu X, Yang Z, Shen H, Liu L, Yu Y (2020). Levodopa improves cognitive function and the deficits of structural synaptic plasticity in hippocampus induced by global cerebral ischemia/reperfusion injury in rats. Front Neurosci.

[30] Feng C, Wan H, Zhang Y, Yu L, Shao C, He Y (2020). Neuroprotective effect of Danhong injection on cerebral ischemia-reperfusion injury in rats by activation of the PI3K-Akt pathway. Front Pharmacol.

