# Supplementary material for: Neuroprotective effects of alisol A 24-acetate on cerebral ischaemia–reperfusion injury are mediated by regulating the PI3K/AKT pathway
Source: J Neuroinflammation. 2022 Feb 7;19:37. doi: 10.1186/s12974-022-02392-3 (PMC8822821; doi:10.1186/s12974-022-02392-3)
Supplement: Supplementary file 1 — Additional file 1. Monitoring of cerebral blood flow and detection of brain tissue ischemia in mice. [file 12974_2022_2392_MOESM1_ESM.docx]

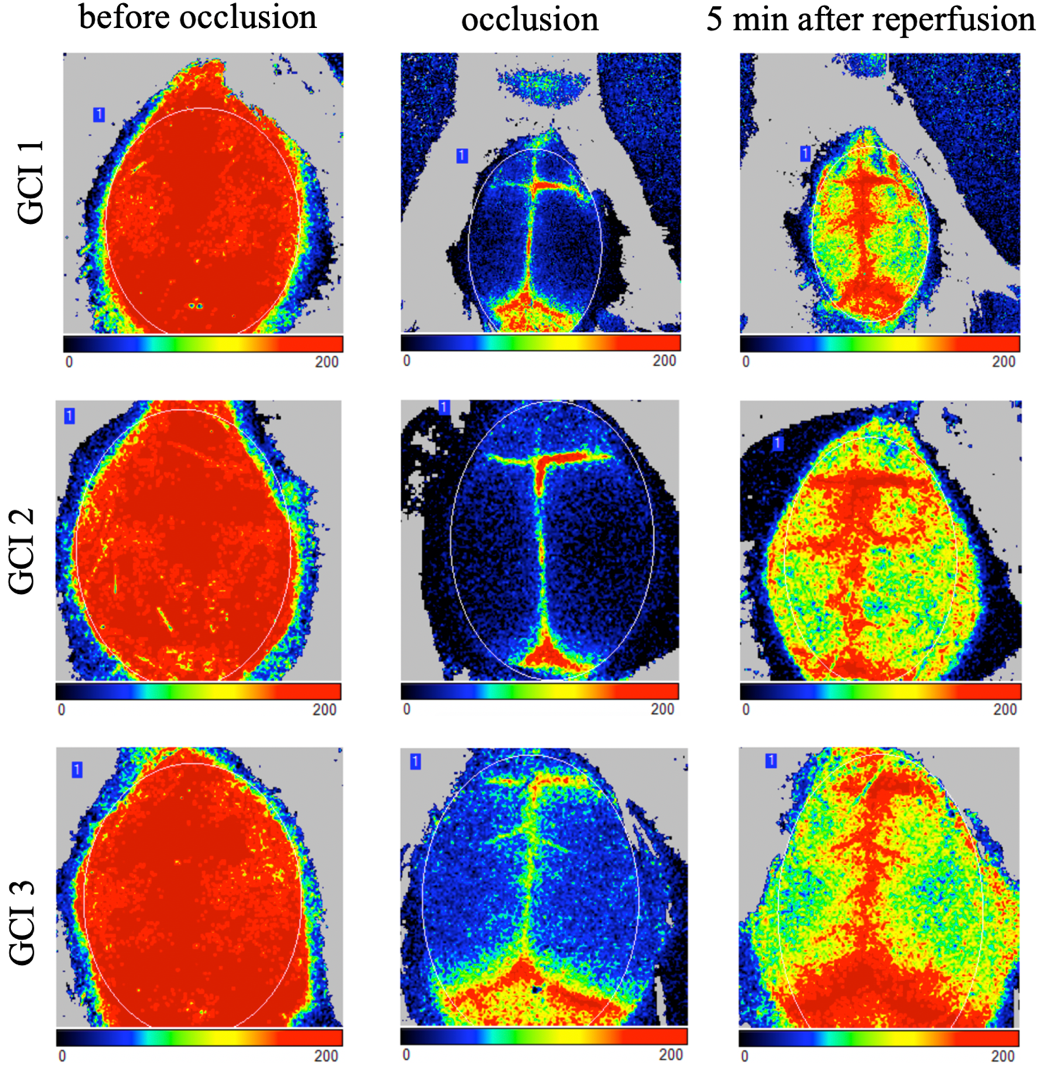


**Fig. S1** **Cerebral blood flow of the mice monitored by laser speckle.** Using laser speckle imaging, which can visualize quadratic distribution of cerebral blood flow (CBF), we measured blood flow changes in transient cerebral ischemic mice. We found that CBF of the mice was significantly decreased, and there was no obvious compensation in the posterior circulation after the bilateral common carotid arteries were closed. 20 minutes later, the surgical thread was untied, and laser speckle was used to detect CBF again at 5 minutes after reperfusion.

**Fig. S2** **T2-weighted imaging of mice brain after ischemia-reperfusion for 48
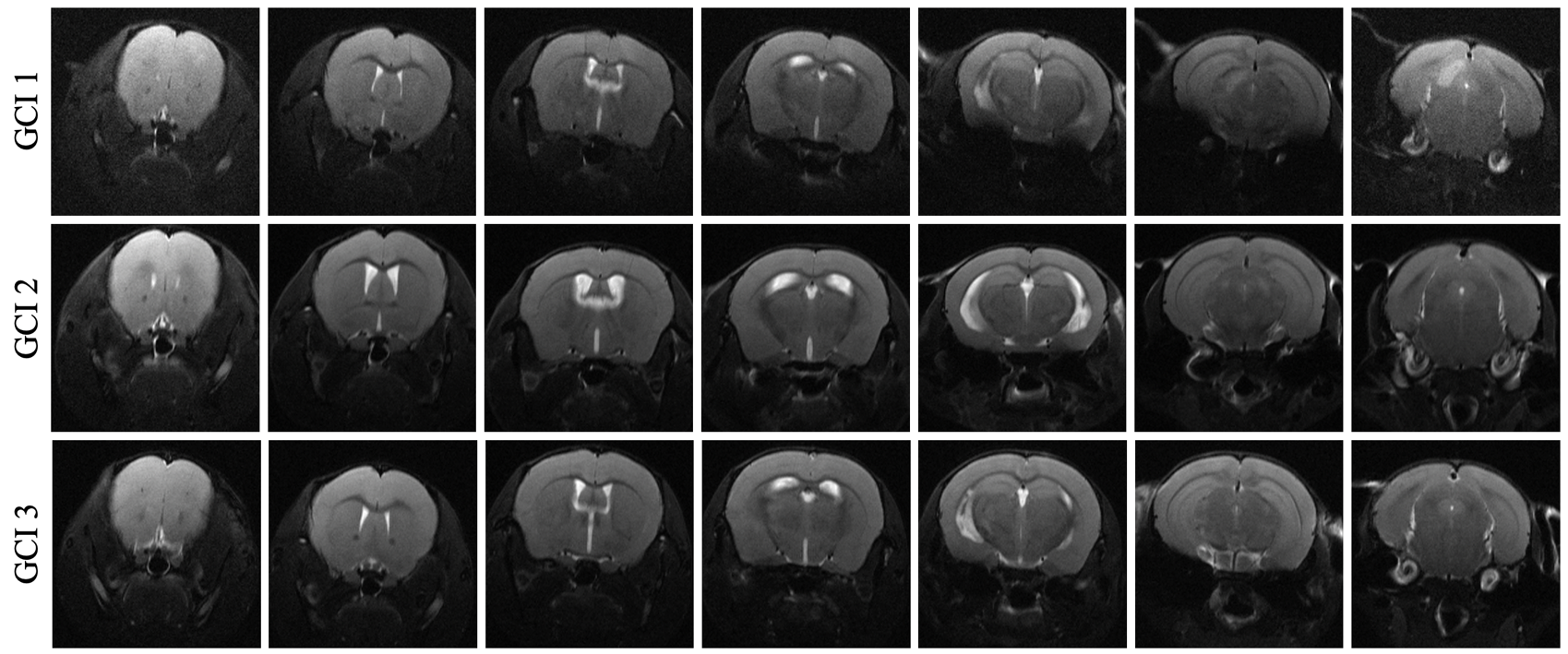
h.** 48 hours after reperfusion, MRI scans (T2-weighted imaging) were used to observe the brain tissue ischemia in mice. After MRI, the changes of MRI signals in the mouse brain were observed, and a clinician with 5 years' clinical experience independently measured the signal values on T2WI images. The results showed that transient global cerebral ischemia model did not caused infarcts in specific brain tissues in the present study.
